# Black TiO_2_-Based Dual Photoanodes Boost the Efficiency of Quantum Dot-Sensitized Solar Cells to 11.7%

**DOI:** 10.3390/nano12234294

**Published:** 2022-12-02

**Authors:** Danwen Yao, Zhenyu Hu, Ruifeng Zheng, Jialun Li, Liying Wang, Xijia Yang, Wei Lü, Huailiang Xu

**Affiliations:** 1 State Key Laboratory of Integrated Optoelectronics, College of Electronic Science and Engineering, Jilin University, Changchun 130012, China; 2 State Key Laboratory of Advanced Structural Materials, Ministry of Education, Changchun University of Technology, Changchun 130012, China; 3 State Key Laboratory of Precision Spectroscopy and Chongqing Institute, East China Normal University, Shanghai 200062, China

**Keywords:** black TiO_2_, dual photoanode, quantum dot-sensitized solar cell

## Abstract

Quantum dot-sensitized solar cells (QDSSC) have been regarded as one of the most promising candidates for effective utilization of solar energy, but its power conversion efficiency (PCE) is still far from meeting expectations. One of the most important bottlenecks is the limited collection efficiency of photogenerated electrons in the photoanodes. Herein, we design QDSSCs with a dual-photoanode architecture, and assemble the dual photoanodes with black TiO_2_ nanoparticles (NPs), which were processed by a femtosecond laser in the filamentation regime, and common CdS/CdSe QD sensitizers. A maximum PCE of 11.7% with a short circuit current density of 50.3 mA/cm^2^ is unambiguously achieved. We reveal both experimentally and theoretically that the enhanced PCE is mainly attributed to the improved light harvesting of black TiO_2_ due to the black TiO_2_ shells formed on white TiO_2_ NPs.

## 1. Introduction

In recent decades, energy shortages and environmental pollution have emerged as some of the most important concerns that need to be addressed [1]. Solar cells are an effective strategy to utilize solar energy as a kind of clean energy [2]. Among the third-generation solar cells, quantum dot-sensitized solar cells (QDSSCs) have attracted considerable attention due to the stunning properties of quantum dots (QDs) such as a tunable bandgap, a large light absorption coefficient, high stability, and multiple exciton effects [3,4]. Typical QDSSCs are composed of a photoanode, an electrolyte, and a counter electrode (CE). The photoanode is the crucial part and is responsible for the collection and transfer of photogenerated electrons, and generally consists of a mesoporous, wide bandgap oxide film (electron acceptor) coated with a light harvesting material (QD sensitizer). An ideal photoanode should have high specific surface area to absorb QD sensitizers, a suitable crystal structure to obtain extensive light absorption, and excellent ability for electron injection and transfer while having a small charge recombination rate [5]. Although numerous wide bandgap semiconductors such as ZnO, SnO_2_, and ZnS have been used as photoanodes in QDSSCs, TiO_2_ is still the most extensively used material due to its excellent chemical stability, non-toxicity, and low cost, etc. [6,7,8]. Limited by the wide bandgap, TiO_2_ can only absorb ultraviolet light, which accounts for ~5% of sunlight. Although the absorption range of TiO_2_ can be expanded by combining it with narrow bandgap semiconductors such as CdS, CdSe, PbS, CdSe, etc., the power conversion efficiency (PCE) of QDSSCs is still far from the theoretical value in spite of the great efforts dedicated to optimizing the species of narrow bandgap semiconductors and the interface between TiO_2_ and narrow bandgap semiconductors. The highest efficiency of QDSSCs reported so far is 15.31% by Zhong et al., which is based on an elegantly designed Zn–Cu–In–S–Se QD sensitizer [9].

Black TiO_2_ was first discovered and reported by Chen et al. in 2011. Compared with traditional TiO_2_, black TiO_2_ has a higher light absorption intensity and a wider light absorption range, which can be extended to the visible and near-infrared region with lower impedance, and thus has attracted attention in the fields of photodegradation and hydrogen production, etc. [10,11]. In the following several years, numerous researchers tried to fabricate black TiO_2_ by doping S, Se, N, P, and other elements and the final products obtained could be yellow, blue, and black [12,13]. Some researchers inferred that the color change in TiO_2_ NPs may be related to the increase in the oxygen vacancy concentration, leading to a narrower bandgap and redshift in the absorption spectrum to the visible and even the infrared region [14,15,16]. In contrast, some researchers believe that the increase in oxygen vacancy concentration and the doping of other elements would not narrow the bandgap of TiO_2_, and the significant redshift in the absorption spectrum is owing to the amorphous surface state of black TiO_2_ [17,18,19]. In spite of the argument around the mechanism of black TiO_2_, including the effects of oxygen vacancies, Ti^3+^, and doped elements [20,21,22,23], it is clear that black TiO_2_ exhibits enhanced and extended light absorption, which is expected to improve the performance of QDSSCs [24,25,26,27].

Herein, we fabricate black TiO_2_ by femtosecond laser treatment of commercial TiO_2_ nanoparticles (NPs). The black TiO_2_ NPs prepared from anatase, rutile, and P25 (mixed-phase TiO_2_ crystal with the anatase/rutile ratio of ~80:20) are further used to assemble QDSSCs. A solar cell architecture combining the concentrated photovoltaic cell (CPV) concept with the design of a dual photoanode is proposed, where CuS is used as the CE and sandwiched between two black TiO_2_ photoanodes. A PCE of 11.7% with a recorded *J_sc_* of 50.3 mA/cm^2^ is achieved, showing a 290% enhancement when compared with that assembled by traditional P25 photoanodes. According to theoretical and experimental analyses, the PCE improvement is attributed to the black TiO_2_ shells formed on white TiO_2_ NPs, which expand the absorption of black TiO_2_ to the visible and infrared region and induce more excited photoelectrons in the photoanode. In addition, the dual photoanode design and CPV integration enable the augmented light to transmit to the bottom and top photoanodes, providing additional excitation energy for light harvesting.

## 2. Materials and Methods

### 2.1. Preparation of Black TiO_2_

**Figure 1:**
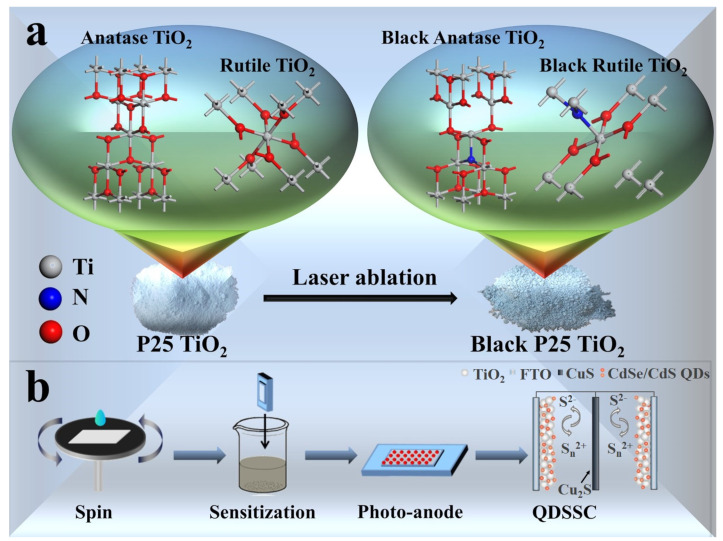
(**a**) Schematic illustration of structures of pristine and black TiO_2_; (**b**) Fabrication procedure and working principle of QDSSCs.

Black P25, rutile TiO_2_, and anatase TiO_2_ were prepared by a titanium–sapphire laser system (Spectra-Physics, Spitfire ACE), which generated linearly polarized femtosecond laser pulses with a center wavelength of 800 nm, a repetition rate of 500 Hz, and a pulse width of about 40 fs [27]. A powdered sample of TiO_2_ was flattened on the bottom of the container with a glass slide, and the container was fixed on a two-dimensional movable platform equipped with electric motors. A rotatable half-wave plate and a polarizer were placed in the laser propagation path to control the laser pulse energy at 1.0 mJ. The laser beam was then reflected by two mirrors with high reflection at 800 nm to make the beam propagate vertically to the surface of the TiO_2_ samples. The laser beam passed through a *f* = 1 m focal lens to form a single filament [28,29,30], and then hit the flattened TiO_2_ sample to produce black TiO_2_. The container was moved with the electric motors so as to raster it. As an example, the pristine and processed P25 TiO_2_ samples are shown in Figure 1a with their crystalline structures.

### 2.2. Preparation of Photoanodes

Typically, 0.8 g TiO_2_ (Aladdin, Shangai, China), 0.4 g ethyl cellulose (Aladdin), and 3.245 g α-terpineol (Aladdin) were dispersed in 8.5 mL ethanol (Sinopharm Chemical Reagent Co., Ltd., Shanghai, China) under stirring. The obtained paste was spin-coated on FTO substrates (Zhuhai Kaiwo Optoelectronics Technology Co., Ltd., Zhuhai, China) and dried at 80 °C for 20 min, where the FTO substrate had been cleaned by immersing in deionized water, absolute ethanol, acetone, absolute ethanol, and deionized water in turn and washing by ultrasonic methods for 30 min, respectively. Then, the films were annealed at 450 °C for 30 min to acquire porous TiO_2_ films. The prepared TiO_2_ film was sensitized with CdS QDs by the successive ionic layer adsorption and reaction method using cadmium acetate (0.05 M, Sinopharm Chemical Reagent Co., Ltd.) and anhydrous sodium sulfite (0.05 M, Aladdin). Then, the chemical bath deposition method was used to deposit CdSe QDs using selenium powder (0.08 M, Tianjin Guangfu Science and Technology Seven Development Co., Ltd., Tianjin, China), Na_2_SO_3_ (0.05 M, Aladdin), Cd(CH_3_COO)_2_ (0.05 M, Aladdin), and trisodium nitrilotriacetic acid monohydrate (0.12 M, TCI Shanghai). Finally, the ZnS passivation layer was coated by immersing the photoanode in 0.1M Zn(AC)_2_·2H_2_O solution (Sinopharm Chemical Reagent Co., Ltd.) and 0.1 M Na_2_S·9H_2_O (Aladdin) solution repeatedly for two cycles.

### 2.3. Preparation of CuS and CuS/Brass-Mesh Counter Electrode (CE)

To prepare CuS CEs, a 50 mL solution including Na_2_S_2_O_3_·5H_2_O (1 M) and CuS·5H_2_O (1 M, Tianjin Guangfu Science and Technology Seven Development Co., Ltd.) was prepared and the pH was adjusted to 2.0 by acetic acid (Aladdin). Clean FTO substrates were put in the solution and kept at 70 °C for 3 h. Then, the substrates were dried at 130 °C for 30 min to acquire CuS CEs. As for the preparation of the CuS/brass-mesh CE, the copper mesh (Hebei Xingheng Materialtech Co., Ltd., China) was soaked in 70 °C hydrochloric acid (36%, Sinopharm Chemical Reagent Co., Ltd.) for 2 h to remove the zinc on the surface. Then, the copper mesh was washed with deionized water and dried at room temperature. The treated Cu mesh was soaked in a mixed solution of CH_4_N_2_S (0.01 M, Sinopharm Chemical Reagent Co., Ltd.) and C_2_H_8_N_2_ (Ethylenediamine, 1.5 M, Tianjin Guangfu Science and Technology Seven Development Co., Ltd.) for 24 h, and then the previous cleaning and drying steps were repeated.

### 2.4. Assembly of QDSSCs and Dual Photoanode QDSSCs

The polysulfide electrolyte was prepared by dissolving 2.4 g Na_2_S (Aladdin) and 0.32 g S powder (Sinopharm Chemical Reagent Co., Ltd.) in a 10 mL solution with a methanol/deionized water volume ratio of 7:3. Finally, the CuS CE, polysulfide electrolyte, and different photoanodes were assembled into a sandwich structure device divided by a polymer gasket filled with polysulfide electrolyte. The CPV concept was integrated into QDSSCs with dual-photoanode architecture (D-A), where the prepared CuS/brass mesh CE mentioned in Section 2.3 was used as the counter electrode and sandwiched between two identical photoanodes. Each photoanode and counter electrode were separated by the aforementioned polymer gasket. The fabrication procedure of QDSSCs is shown in Figure 1b.

### 2.5. Characterization and Electrochemical Measurement

The materials and devices were characterized, respectively, by X-ray diffractometry (XRD, Rigaku X-ray diffractometer, Rigaku, Tokyo, Japan), field emission scanning electron microscopy (FESEM, S4800, Hitachi, Tokyo, Japan), transmission electron microscopy (TEM, FEI Talos F200, FEI, Thermo Scientific, Waltham, MA, USA), ultraviolet/visible-near infrared spectrophotometry (UV-3150), an electrochemical workstation (CHI660C, Shanghai Chenhua Instrument Co., Ltd., Shanghai, China), and by X-ray and ultraviolet photoelectron spectroscopy (XPS and UPS) (Thermo Scientific, Waltham, MA, USA). The photovoltaic performances (J–V curves) were measured by a Keithley 2400 source meter under illumination of an AM 1.5 G solar simulator (Zolix Instruments Co., Ltd., Beijing, China).

## 3. Results

**Figure 2:**
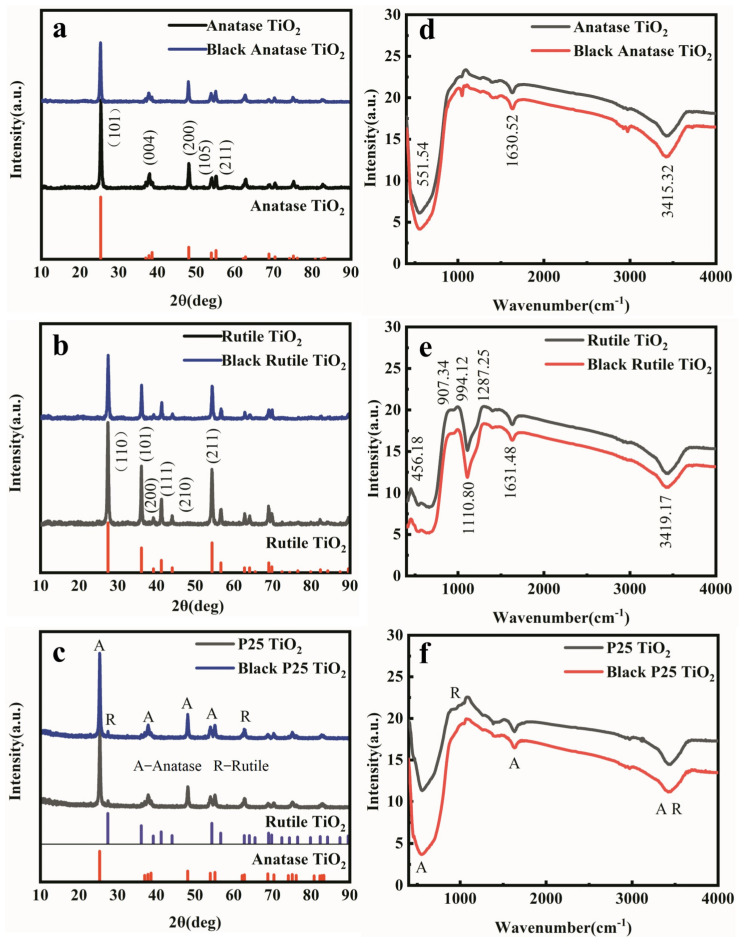
(**a**) XRD patterns of anatase TiO_2_ and black anatase TiO_2_; (**b**) XRD patterns of rutile TiO_2_ and black rutile TiO_2_; (**c**) XRD patterns of P25 and black P25; (**d**) FTIR spectra of anatase TiO_2_ and black anatase TiO_2_; (**e**) FTIR spectra of rutile TiO_2_ and black rutile TiO_2_; (**f**) FTIR spectra of P25 and black P25. A and R in (**c**) and (**f**) represent the peaks from anatase and rutile phases, respectively.

To investigate the effect of phase structure on device performance, three different black TiO_2_ NPs have been prepared based on pristine P25, rutile TiO_2_, and anatase TiO_2_, respectively. The X-ray diffraction (XRD) results in Figure 2a–c indicate that the main diffraction peaks do not change after the laser ablation treatment for all three samples. The peak intensities of anatase and rutile TiO_2_ are weakened slightly, which may be due to the transformation of the surface of TiO_2_ into amorphous TiO_2_, resulting in a decrease in crystallinity, while the decrease in P25 TiO_2_ is almost invisible. Fourier transform infrared spectra (FTIR) are shown in Figure 2d–e. Similar to the XRD results, the infrared peak positions of pristine TiO_2_ are the same as those of black TiO_2_. The characteristic peaks of anatase TiO_2_ and rutile TiO_2_ can be observed in FTIR spectra of P25 TiO_2_ and black P25 TiO_2_ in Figure 2f. In the Raman spectra (Figure S1 in the Supplementary Materials), it can be seen that there are no significant shifts in the Raman peak positions for the black TiO_2_, while the bands seem slightly smaller than the pristine TiO_2_ in all three cases. This indicates that there is no significant difference between black TiO_2_ and white TiO_2_ in structure and functional groups [17,18,19].

**Figure 3:**
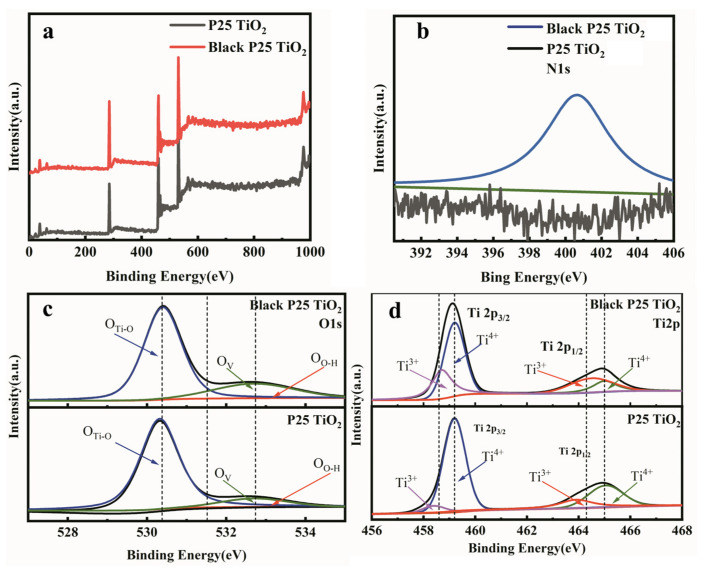
(**a**) XPS survey of P25 TiO_2_ and black P25 TiO_2_; (**b**) N1s XPS spectra of P25 TiO_2_ and black P25 TiO_2_; (**c**) O1s XPS spectra of P25 TiO_2_ and black P25 TiO_2_; (**d**) Ti2p XPS spectra of P25 TiO_2_ and black P25 TiO_2_.

The structural variation was further investigated by X-ray photoelectron spectroscopy (XPS). Figure 3a shows the XPS surveys of P25 TiO_2_ with and without the laser treatment, indicating that there is no obvious difference in their chemical compositions. High-resolution XPS spectra are used to further clarify the detailed variations. The N1s XPS spectrum of black P25 TiO_2_ in Figure 3b indicates that laser treatment of P25 TiO_2_ in air can induce N doping [10,11,13]. Figure 3c shows the O1s XPS spectra of pristine P25 TiO_2_ and black P25 TiO_2_. The peaks at 530.3 eV, 532.2 eV, and 533.2 eV correspond to O_Ti-O_, O_V_, and O_O-H_ bonds, respectively. The oxygen vacancy related peak is located at 532.2 eV, and it can be observed that the O_V_ peak intensity in black P25 TiO_2_ is higher than that in pristine P25 TiO_2_, indicating that the oxygen vacancy after the laser treatment is significantly increased. This is an important reason for the color change of P25 TiO_2_ after the laser treatment. Figure 3d demonstrates the Ti2p XPS spectra of pristine P25 TiO_2_ and black P25 TiO_2_. The two characteristic peaks of Ti^4+^ are located at 459.25 eV and 465.1 eV, and the two characteristic peaks of Ti^3+^ are located at 458.2 eV and 463.95 eV. The proportion of Ti^3+^ in black P25 TiO_2_ is higher than that of pristine P25 TiO_2_, which is consistent with the change of oxygen vacancy content in Figure 3b, confirming the aforementioned explanation [31,32,33].

**Figure 4:**
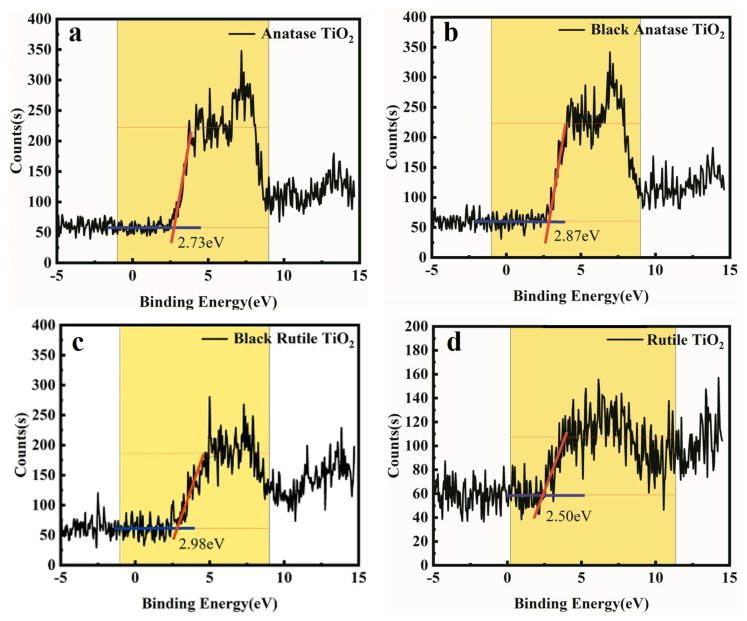
UPS spectra of anatase TiO_2_ (**a**) and black anatase TiO_2_ (**b**); UPS spectra of rutile TiO_2_ (**c**) and black rutile TiO_2_ (**d**). The blue and red lines are the fits to be tangent to the regression curves.

**Figure 5:**
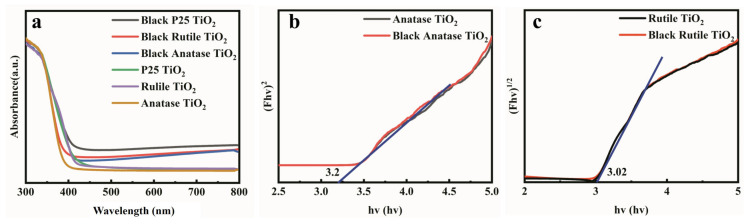
(**a**) The UV–vis absorption spectra of six samples of P25 TiO_2_, rutile TiO_2_, anatase TiO_2_, black P25 TiO_2_, black rutile TiO_2_, and black anatase TiO_2_; (**b**) Plots of (Ahν)^2^ vs. photon energy of anatase TiO_2_ and black anatase TiO_2_; (**c**) Plots of (Ahν)^2^ vs. photon energy of rutile TiO_2_ and black rutile TiO_2_. The blue lines are the fits to be tangent to the experimental curves.

Shown in Figure 4 are the UPS spectra of anatase TiO_2_ and rutile TiO_2_ before and after laser treatment. Both results indicate that the top of the valence band of black TiO_2_ is lower than that of pristine TiO_2_, which is consistent with previous reports [14,15,18]. From the UV–vis absorption spectra (Figure 5a) and diffuse reflectance spectra (Figure S2b in the Supplementary Materials) of the six samples it can be seen that the main absorption of the six samples occurs in the spectral range of less than 400 nm, mainly due to the inherent bandgap of TiO_2_ (3.02–3.20 eV) [34]. It should be emphasized that the change in the absorption up to 2500 nm can also be observed (see Figure S2a in the Supplementary Materials), which indicates the strong effect of the laser treatment on the optical properties of TiO_2_.

The absorption of black TiO_2_ in the visible and infrared region is overall higher than that of pristine TiO_2_, and the significant enhancement in light absorption will generate more photoexcited electrons, and thus a higher photocurrent *J_sc_*. Shown in Figure 5b,c are the bandgap diagrams of anatase TiO_2_ and black anatase TiO_2_, and rutile TiO_2_ and black rutile TiO_2_, respectively, from which it can be seen that the bandgap of the black TiO_2_ has no obvious narrowing. This is in contrast to the black TiO_2_ calcined at high temperature in the reducing gas, in which the reduction of TiO_2_ is comparatively complete and there is a more obvious bandgap narrowing. The laser treatment only acts on the surface of TiO_2_ NPs. The test thickness of UPS is about 1–2 nm; therefore, UPS spectra mainly reflect the surface properties of NPs. Therefore, the black TiO_2_ we prepared is likely to be a core-shell structure, the surface layer should be amorphous TiO_2_, and the core is untreated crystalline TiO_2_. The XPS results of anatase TiO_2_ and rutile TiO_2_ before and after the laser treatment are similar to that of P25 (see Figures S3 and S4 in the Supplementary Materials). The detailed analysis of the energy band variation induced by the laser treatment is further investigated by first-principle calculations and will be shown later.

**Figure 6:**
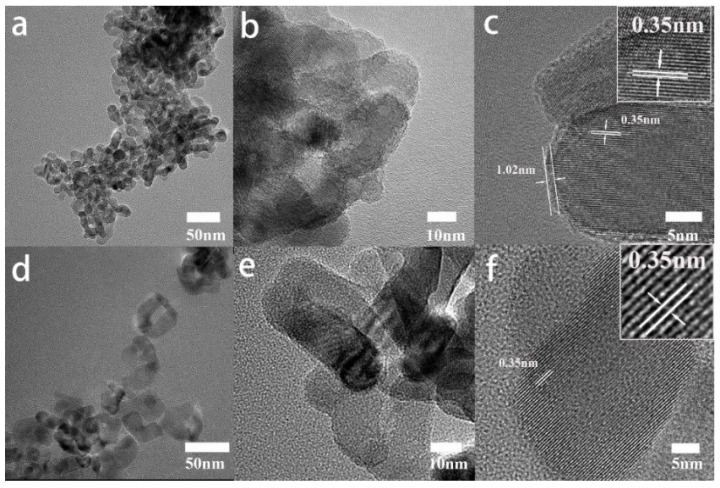
(**a**–**c**) are TEM and HRETM images of black P25 TiO_2_; (**d**–**f**) are TEM and HRETM images of P25 TiO_2_ without laser treatment. Insets in (**c**,**f**): Zoomed-in views for the TEM images.

Figure 6a–c show the TEM images of black P25 TiO_2_, and Figure 6d–f show the TEM images of pristine P25 TiO_2_. It can be seen in Figure 6 that the size of P25 does not change significantly before and after the laser treatment. Both the high-resolution TEM (HRTEM) images shown in Figure 6c,f show a clear lattice spacing of 0.35 nm, which corresponds to the (101) plane of TiO_2_. The results are consistent with the aforementioned prediction that the core is untreated crystalline TiO_2_, and the difference exists on the surface area. For black P25, an amorphous layer with 1.02 nm thickness can be observed. That is to say, the black TiO_2_ prepared by the laser treatment is a core-shell structure, the surface layer is amorphous TiO_2_, and the core is untreated crystalline TiO_2_, which confirms the conclusion drawn from Figure 3, Figure 4 and Figure 5. Moreover, the TEM images of anatase TiO_2_ and rutile TiO_2_ before and after the laser treatment (see Figures S5–S8 of the Supplementary Materials) give the same results as those in Figure 6.

**Figure 7:**
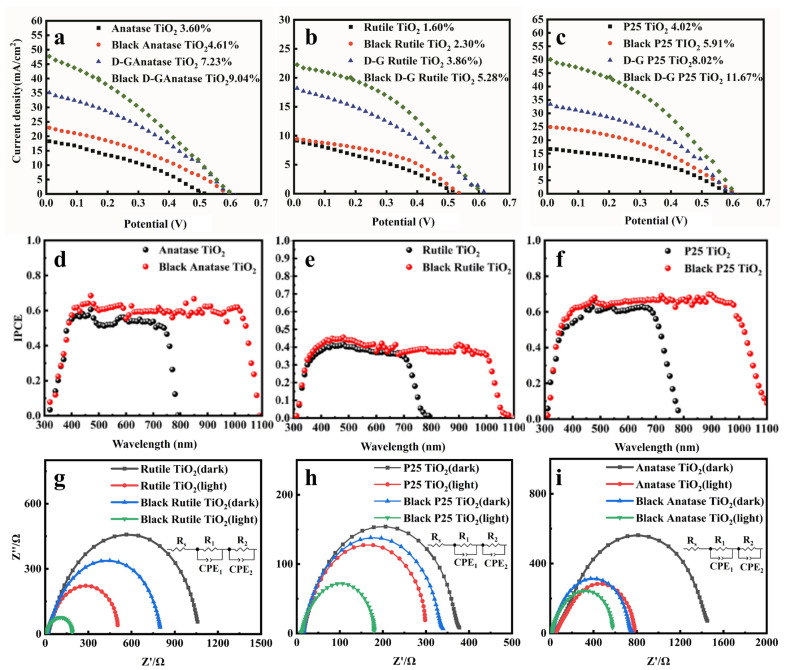
(**a**–**c**) are J–V curves of QDSSCs based on anatase TiO_2_, rutile TiO_2_, and P25 TiO_2_ photoanodes with and without laser treatment, respectively; (**d**–**f**) are IPCE curves of QDSSCs based on anatase TiO_2_, rutile TiO_2_, and P25 TiO_2_ photoanodes with and without laser treatment, respectively; (**g**–**i**) are Nyquist curves of anatase TiO_2_, rutile TiO_2_, and P25 TiO_2_ photoanodes with and without laser treatment measured in the dark and under illumination, respectively.

**Figure 8:**
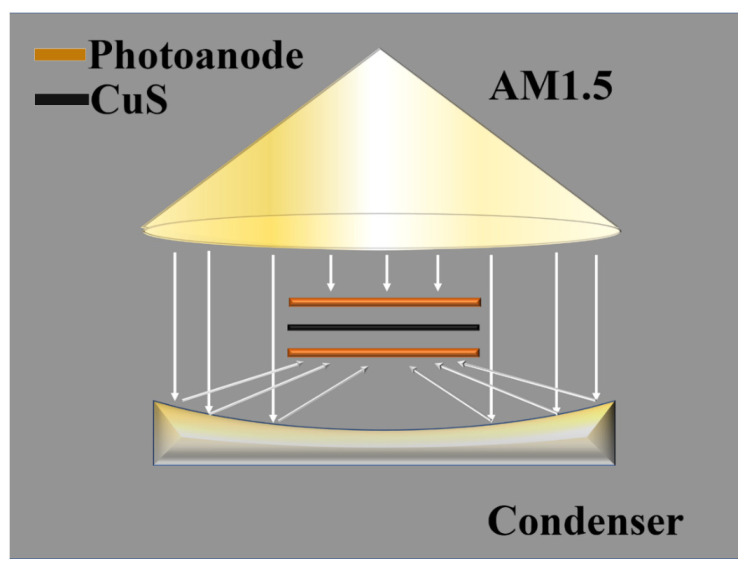
Schematic diagram of the cell design with dual photoanode used in the present work.

Then, we investigated the PCEs of QDSSCs fabricated with a single TiO_2_ photoanode, and the corresponding device parameters are shown in Table 1. For J–V curves of anatase TiO_2_ in Figure 7a, the PCE of anatase TiO_2_ is 3.6% with a *J_sc_* = 18.3 mA/cm^2^, and for black anatase TiO_2_ the PCE is 4.7% with a *J_sc_* = 22.9 mA/cm^2^. The overall PCE is increased by 30%, which is due to the improved absorption of TiO_2_ after the laser treatment. The schematic diagram of the dual photoanodes is shown in Figure 8. For the PCE of D-A TiO_2_ QDSSCs, by one sun irradiating from the top photoanode and with concentrated sunshine illumination from the bottom photoanode with the help of a parabolic reflector, the PCE is found to be greatly enhanced. The PCE of dual anatase TiO_2_ photoanode is 7.2%, and that of black anatase TiO_2_ is 9.1%. For rutile TiO_2_ in Figure 7b, the PCE increased from 1.6% to 2.3% after the laser treatment, and the overall PCE increased by 43.75%. The PCE of the dual rutile TiO_2_ photoanode is 3.9% and that of the black rutile TiO_2_ is 5.3%. The best performance is achieved by P25 TiO_2_ and is shown in Figure 7c, which is due to the synergic effect of rutile and anatase TiO_2_. The *J_sc_* increased from 16.6 mA/cm^2^ to 25.0 mA/cm^2^ after the laser treatment, inducing an increasing in PCE from 4.0% to 5.9%. The PCE of the dual P25 TiO_2_ photoanode is 8.0% and that of the black P25 TiO_2_ is 11.7% with a *J_sc_* of 50.3 mA/cm^2^. From the J–V curves, it can be confirmed that the increase in PCE is mainly due to the enhanced *J_sc_*, which suggests the improved collection efficiency of photogenerated electrons.

To further verify the above mechanism, we measured incident photon-to-electron conversion efficiency (IPCE), as shown in Figure 7d–f. For pristine TiO_2_ without the laser treatment, all three kinds of devices exhibit a photo-response in the visible region, which is consistent with previous reports since CdS/CdSe QD sensitizers absorb visible light. Replacing pristine TiO_2_ with black TiO_2_ induces the extension of the IPCE to the near-infrared region as shown by the red dotted curves, which is responsible for the increase in *J_sc_*. It should be noticed that the QD sensitizers used in the present study only absorb visible light, and thus the extended IPCE should be caused by the black TiO_2_, which is consistent with the absorption properties of black TiO_2_, shown in Figure S2 in the Supplementary Materials. In addition, it should be mentioned that we adopted a dual photoanode architecture, and two photoanodes are connected in parallel and share a CuS mesh CE. Meanwhile, the CPV concept was integrated into QDSSCs. Light management as an important technology to improve the conversion efficiency in solar cells; aiming to increase the photon flux received by solar cells. By the light trapping effect, the optical path length is increased, thereby improving the PCE of solar cells. The IPCE test on two different measurement devices (Crowntech QTest Station 1000 CE, America and DG-6050 Zolix, China) are different from the PCE test on two different solar simulators (SS150 Solar Simulator Zolix and Sirius-SS150A-D Zolix), where sunlight is illuminated on both sides of cell. This is because the IPCE test only allows illumination from the top side due to the device design. Therefore, the actual IPCE should be higher than the present test value in Figure 7. The reported PCE 11.7% is an average value, and a larger PCE of over 12% could be acquired.

Shown in Figure 7g–i are Nyquist curves of anatase TiO_2_, rutile TiO_2_, and P25 TiO_2_ photoanodes with and without the laser treatment, which were measured in the dark and under illumination, respectively. The Nyquist curves are obtained from the Electrochemical Impedance Spectroscopy (EIS) measurements, which can reveal the interfacial reactions of photoexcited electrons in the QDSSCs. The electrochemical system can be regarded as the equivalent circuit shown in the inset, where R_s_ represents the series resistance in the high frequency region, and CPE_1_ and CPE_2_ are the chemical capacitances of the cathode and photoanode, respectively. R_1_ and R_2_ refer to the charge transfer resistance between the counter electrode and electrolyte (R_1_) and that at the TiO_2_/QDs/electrolyte interface (R_2_), which can be determined from the radii of the first and second circles of the Nyquist curve, respectively. As shown in Figure 7g–i, the radii of all of the first circles of the Nyquist curves are unobservable, indicating that the charge transfer resistance R_1_ is negligible for all the QDSSC devices. Furthermore, the radii of the second circles of the black TiO_2_ based photoanodes are remarkably smaller than those of the pristine TiO_2_, corresponding to the smaller charge transfer resistance, which indicates that the charge transport of black TiO_2_-based devices is faster than that of pristine TiO_2_-based photoanodes. This is due to oxygen vacancy doping providing a more convenient transport channel for electrons [8,35]. This greatly improves the ability of the TiO_2_ thin film to separate, collect, and transport photogenerated carriers in the cell, thereby reducing the loss of the photogenerated carriers during the device operation. Among the three kinds of TiO_2_, the smallest impedance is achieved by black P25 TiO_2_, which is consistent with the J–V results. Compared with the pristine sample, the decrease in interfacial electron transfer resistance enables facilitation of electron transport, leading to a significant increase in *J_sc_*.

**Figure 9:**
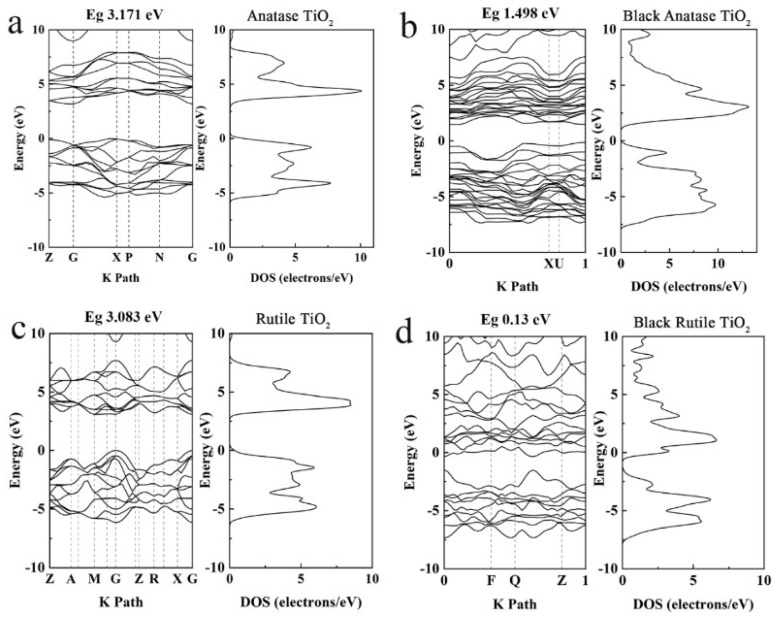
Energy band and density of states diagram based on first-principle calculation for (**a**) anatase TiO_2_, (**b**) black anatase TiO_2_ rich in oxygen vacancies, (**c**) rutile TiO_2_, and (**d**) black rutile TiO_2_ rich in oxygen vacancies.

XPS has confirmed that the laser processing can induce oxygen vacancies accompanied by N-doping in TiO_2_, while TEM results suggest that the laser processing only affects the surface of TiO_2_ NPs, inducing a core/shell structure; thus, the oxygen vacancies and N-doping should mainly exist in the surface layer. The results in Figure 2 indicate that the bandgap does not change obviously before and after the laser treatment, which is believed to originate from the measurement depth of XPS, and reflects the information on near surface layer. Therefore, the analysis of energy band variation induced by the laser processing is further investigated by first-principle calculation of pristine TiO_2_ and N-doped TiO_2_ rich in oxygen vacancies induced by laser processing [28,30,36,37,38,39]. It is found that the oxygen atoms in the anatase phase are equivalent, so any oxygen vacancies will not affect the calculation results. Shown in Figure 9 are the calculated results in the range of −10–10 eV for anatase and rutile TiO_2_ before and after the laser treatment (see Figure S9 in the Supplementary Materials for a larger energy range of −60–30 eV). For anatase TiO_2_, it can be observed from Figure 9a,b that the bandgap of TiO_2_ has been significantly narrowed due to the doping of oxygen vacancies and nitrogen atoms. This is mainly due to the donor behavior of the introduced oxygen vacancies, leading to the formation of a donor level. Thus, the forbidden band width is reduced from 3.171 eV to 1.498 eV. However, the substitutional doping of O atoms by N atoms would introduce holes, which act as acceptors and produce a shallow acceptor energy level above the top of the valence band of pristine anatase TiO_2_; therefore, there is no sharp narrowing in the bandgap. For rutile TiO_2_ in Figure 9c,d, similar bandgap narrowing from 3.083 to 0.13 eV can be confirmed. This is significantly larger than that of anatase TiO_2_. Compared with the anatase TiO_2_ structure, the rutile unit cell has fewer atoms (see Figure S10 in the Supplementary Materials for unit cells of the pristine TiO_2_ and black TiO_2_ with nitrogen-doping and oxygen vacancies). The same number of oxygen vacancies and nitrogen atoms account for a larger atomic proportion, which should be the reason for the smaller calculated bandgap than that of anatase TiO_2_. The experimental and theoretical investigations in the present work have shown that the laser processing mainly induces N-doping and oxygen vacancies near the surface layer of TiO_2_, which reduces the bandgap, inducing a broadened absorption [40].

Since black TiO_2_ itself shows broad absorption, it is possible to use black TiO_2_ as a photoanode, without loading CdS/CdSe sensitizers, to realize high PCE (the corresponding results are shown in Figure S11 of the Supplementary Materials). It could be confirmed that the pure black TiO_2_ photoanode shows very poor PCE, suggesting that the pure black TiO_2_ photoanode cannot realize effective photogenerated electron extraction, which is probably due to a failure to find a suitable electrolyte and CE that matched black TiO_2_. The polysulfide (S^2–^/Sn^2–^) electrolyte is commonly used in QDSSCs since it can stabilize the commonly used chalcogenide QD sensitizers and provide an acceptable photovoltaic performance [41]. However, in the absence of sulfide QDs, the polysulfide electrolyte could not directly combine with TiO_2_ to achieve redox reaction. Photosensitizer can only be used in solar cells if the right electrolyte is combined with a matching pair of electrodes [26].

While the debate around the photoelectrical and photocatalytic mechanism of black TiO_2_ still exists, the improvement in PCE of QDSSCs in this work could be attributed to two reasons according to our experimental and theoretical investigations: (i) the expanded absorption of black TiO_2_ to the near-infrared region induces more excited photoelectrons in the photoanode, and thus increases the *J_sc_*; and (ii) the dual photoanode design and CPV integration, where the two photoanodes are connected in parallel and share a CuS mesh CE. The CuS grown on Cu mesh as a CE allows the transmitted light from the top cell to arrive at the bottom photoanode. Thus, the dual photoanode structure can capture more light and increase current density. In addition, the CPV systems can also enable an augmented light transmission to the top photoanode through the parabolic reflector at the bottom, providing additional excitation energy for light harvesting. To our best knowledge, the present work achieves the highest PCE of CdS/CdSe QD co-sensitized QDSSCs, which is mainly due to the obvious enhancement in *J_sc_* (Table S1 in Supplementary Materials).

## 4. Conclusions

In the present study, we have developed a strategy to boost performance of QDSSCs, in which black TiO_2_ produced by laser processing was used as a photoanode material. It has been shown that the laser fabrication induces N-doping and oxygen vacancies near the surface layer of TiO_2_ and forms the core/shell structure, the synergic effect of which may be the origin for the extension of the absorption of black TiO_2_ into the visible and infrared region. Combined with the CPV structure design, a PCE of 11.7% with a *J_sc_* of 50.3 mA/cm^2^ in the QDSSCs were achieved, which is due to the expanded absorption of black TiO_2_, the dual photoanode design, and CPV integration. While great efforts have been dedicated to synthesizing novel QD sensitizers and to the complicated structure design of photoanodes, the present work has provided an alternative way to boost performance of QDSSCs, which are expected to exhibit higher performance by further optimization of cell design parameters.

## Figures and Tables

**Table 1:** Performance parameters of QDSSCS based on different photoanodes, and D-A means dual photoanodes.

Sample	*J_sc_* (mA/cm^2^)	*V_oc_* (V)	FF	PCE (%)
Rutile TiO_2_	9.2 ± 0.2	0.510 ± 0.003	0.267 ± 0.001	1.6 ± 0.1
Anatase TiO_2_	16.5 ± 0.2	0.569 ± 0.001	0.385 ± 0.003	3.6 ± 0.1
P25 TiO_2_	16.6 ± 0.3	0.594 ± 0.002	0.403 ± 0.004	4.0 ± 0.1
Black Rutile TiO_2_	12.1 ± 0.2	0.542 ± 0.003	0.379 ± 0.003	2.3 ± 0.1
Black Anatase TiO_2_	22.9 ± 0.3	0.607 ± 0.002	0.341 ± 0.006	4.7 ± 0.1
Black P25 TiO_2_	25.0 ± 0.3	0.616 ± 0.004	0.383 ± 0.008	5.9 ± 0.2
D-A Rutile TiO_2_	18.2 ± 0.2	0.634 ± 0.002	0.345 ± 0.004	3.9 ± 0.2
D-A Anatase TiO_2_	34.2 ± 0.3	0.616 ± 0.005	0.342 ± 0.006	7.2 ± 0.1
D-A P25 TiO_2_	33.1 ± 0.4	0.602 ± 0.003	0.405 ± 0.005	8.0 ± 0.2
D-A Black Rutile TiO_2_	22.1 ± 0.4	0.612 ± 0.006	0.398 ± 0.006	5.3 ± 0.2
D-A Black Anatase TiO_2_	47.7 ± 0.4	0.607 ± 0.007	0.326 ± 0.008	9.1 ± 0.3
D-A Black P25 TiO_2_	50.3 ± 0.4	0.619 ± 0.007	0.399 ± 0.007	11.7 ± 0.3

## Data Availability

Data supporting the results presented in this paper are not publicly available at this time but may be obtained from the authors upon reasonable request.

## Supplementary Materials

The following supporting information can be downloaded at: https://www.mdpi.com/article/10.3390/nano12234294/s1. Figure S1: (a) Raman spectra of anatase TiO_2_ and black anatase TiO_2_. (b) Raman spectra of rutile TiO_2_ and black rutile TiO_2_. (c) Raman spectra of P25 TiO_2_ and black P25 TiO_2_, Figure S2: (a) Diffuse reflectance spectra of anatase, rutile, P25, black anatase, black rutile, black P25 nanoparticles. (b) Diffuse absorb spectra of anatase, rutile, P25, black anatase, black rutile, black P25 nanoparticles, Figure S3: (a) O1s XPS spectrum of anatase TiO_2_. (b) Ti2p XPS spectrum of anatase TiO_2_. (c) UPS spectrum of anatase TiO_2_. (d) O1s XPS spectrum of black anatase TiO_2_. (e) Ti2p XPS spectrum of black anatase TiO_2_. (f) UPS spectrum of black anatase TiO_2_. (g) XPS survey of anatase TiO_2_. (h) XPS survey of black anatase TiO_2_. (i) N1s XPS spectra of anatase TiO_2_ and black anatase TiO_2_. The green line is the baseline of the curve, Figure S4: (a) O1s XPS spectrum of rutile TiO_2_. (b) Ti2p XPS spectrum of rutile TiO_2_. (c) UPS spectrum of rutile TiO_2_. (d) O1s XPS spectrum of black rutile TiO_2_. (e) Ti2p XPS spectrum of black rutile TiO_2_. (f) UPS spectrum of black rutile TiO_2_. (g) XPS survey of rutile TiO_2_. (h) XPS survey of black rutile TiO_2_. (i) N1s XPS spectrum of rutile TiO_2_ and black rutile TiO_2_. The green line is the baseline of the curve, Figure S5: TEM and HRTEM images of anatase TiO_2_ with different magnifications (a-f), Figure S6: TEM and HRTEM images of black anatase TiO_2_ with different magnifications (a-f), Figure S7: TEM and HRTEM images of rutile TiO_2_ with different magnifications (a-f), Figure S8: TEM and HRTEM images of black rutile TiO_2_ with different magnifications (a-f), Figure S9: Energy band diagram and density of states spectrum obtained by first-principles calculations for (a) anatase TiO_2_, (b) black anatase TiO_2_, (c) rutile TiO_2_, and (d) black rutile TiO_2_, Figure S10: Unit cells of anatase TiO_2_, black anatase TiO_2_, rutile TiO_2_, and black rutile TiO_2_ for first-principles calculations, Figure S11: (a), (b) and (c) are J-V curves of black anatase TiO_2_, rutile TiO_2_, and P25 TiO_2_ samples assembled with a S^2−^/Sn^2−^ electrolyte and copper sulfide counter electrode without quantum dot sensitization. (d), (e) and (f) are J-V curves of black anatase TiO_2_, rutile TiO_2_, and P25 TiO_2_ samples assembled with a platinum electrode using S^2−^/Sn^2−^ electrolyte without quantum dot sensitization, Table S1: Performance parameters of CdS/CdSe co-sensitized QDSSCs based on different reports. References [42,43,44,45,46,47,48,49,50,51] are cited in the Supplementary Materials.
