# Supplementary material for: Black TiO2-Based Dual Photoanodes Boost the Efficiency of Quantum Dot-Sensitized Solar Cells to 11.7%
Source: Nanomaterials (Basel). 2022 Dec 2;12(23):4294. doi: 10.3390/nano12234294 (PMC9741270; doi:10.3390/nano12234294)
Supplement: Supplementary file 1 [file nanomaterials-12-04294-s001.zip › nanomaterials-2001748-supplementary.pdf]

nanomaterials

Supplementary Materials

Black TiO_2-Based Dual Photoanodes Boost the Efficiency of Quantum Dot-Sensitized Solar Cells to 11.7%

Danwen Yao [1,†], Zhenyu Hu [2,†], Ruifeng Zheng [2], Jialun Li [2], Liying Wang [2], Xijia Yang [2], Wei Lü [2,*] and Huailiang Xu [1,3,*]

1 State Key Laboratory of Integrated Optoelectronics, College of Electronic Science and Engineering, Jilin University, Changchun 130012, China
2 State Key Laboratory of Advanced Structural Materials, Ministry of Education, Changchun University of Technology, Changchun 130012, China
3 State Key Laboratory of Precision Spectroscopy and Chongqing Institute, East China Normal University, Shanghai 200062, China
* Correspondence: lvwei@ccut.edu.cn (W.L.); huailiang@jlu.edu.cn (H.X.).
† These authors contribute equally to this work.

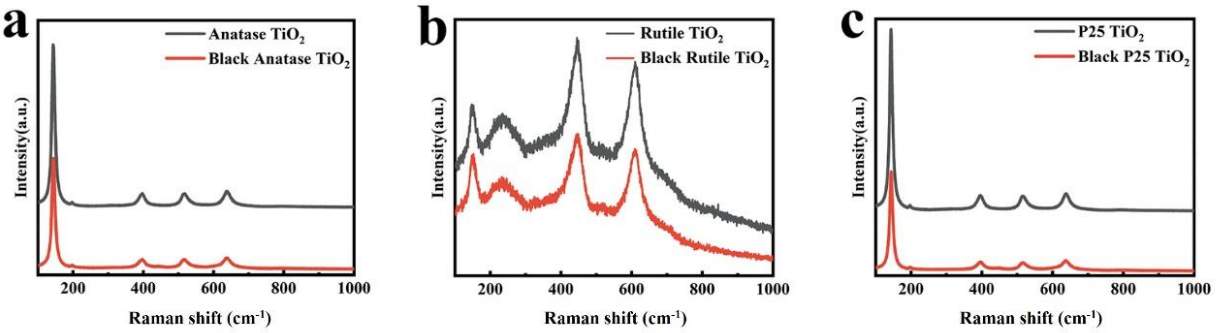

Figure S1. (**a**) Raman spectra of anatase TiO_2 and black anatase TiO_2. (**b**) Raman spectra of rutile TiO_2 and black rutile TiO_2. (**c**) Raman spectra of P25 TiO_2 and black P25 TiO_2.

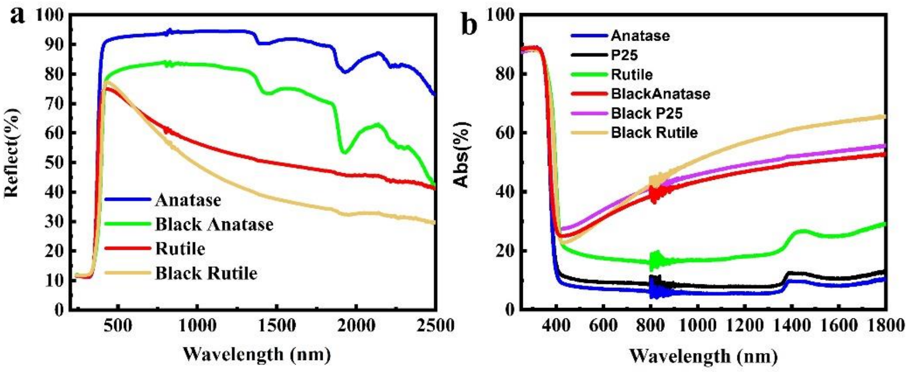

Figure S2. (**a**) Diffuse reflectance spectra of anatase, rutile, P25, black anatase, black rutile, black P25 nanoparticles. (**b**) Diffuse absorb spectra of anatase, rutile, P25, black anatase, black rutile, black P25 nanoparticles.

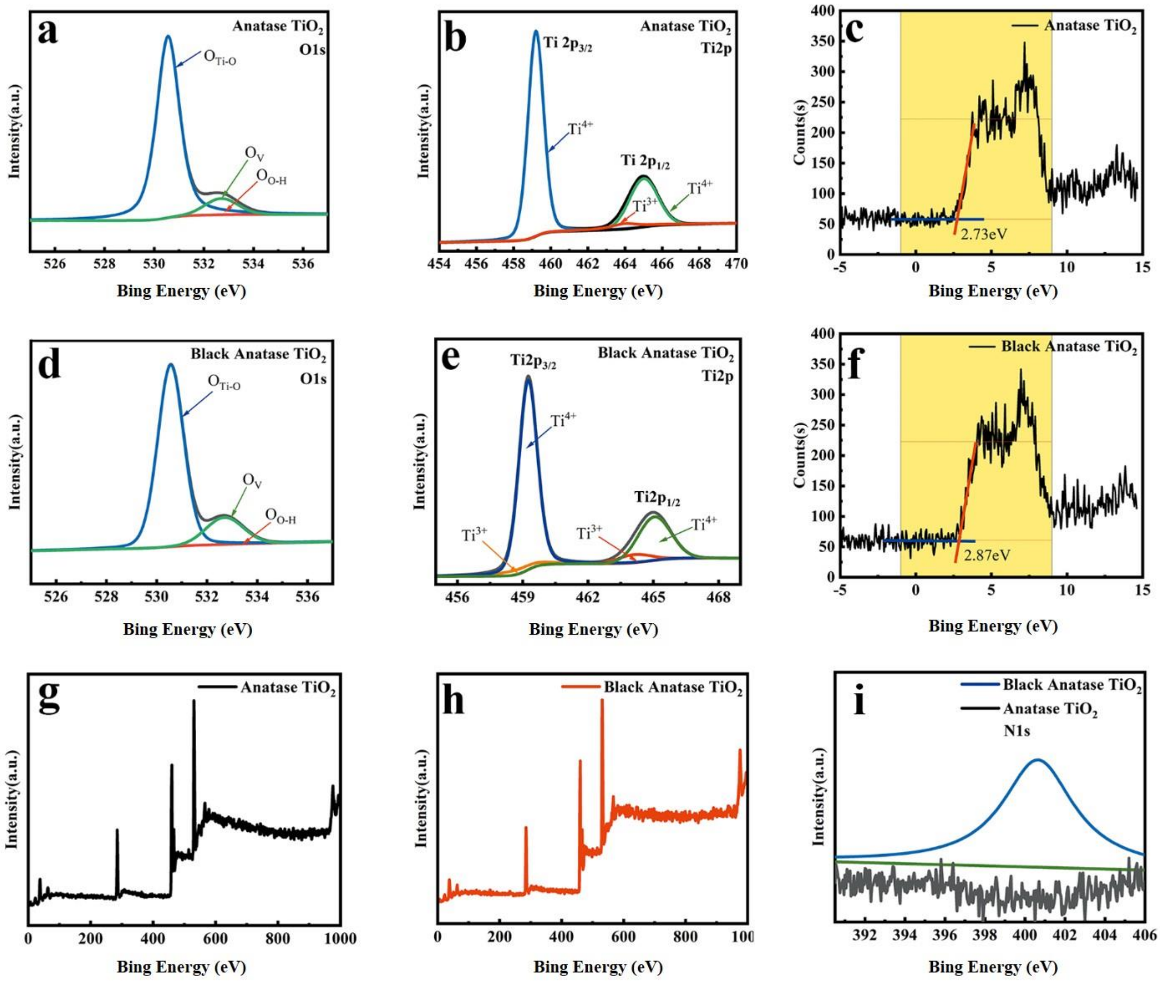

Figure S3. (**a**) O1s XPS spectrum of anatase TiO_2. (**b**) Ti2p XPS spectrum of anatase TiO_2. (**c**) UPS spectrum of anatase TiO_2. (**d**) O1s XPS spectrum of black anatase TiO_2. (**e**) Ti2p XPS spectrum of black anatase TiO_2. (**f**) UPS spectrum of black anatase TiO_2. (**g**) XPS survey of anatase TiO_2. (**h**) XPS survey of black anatase TiO_2. (**i**) N1s XPS spectra of anatase TiO_2 and black anatase TiO_2. The green line is the baseline of the curve.

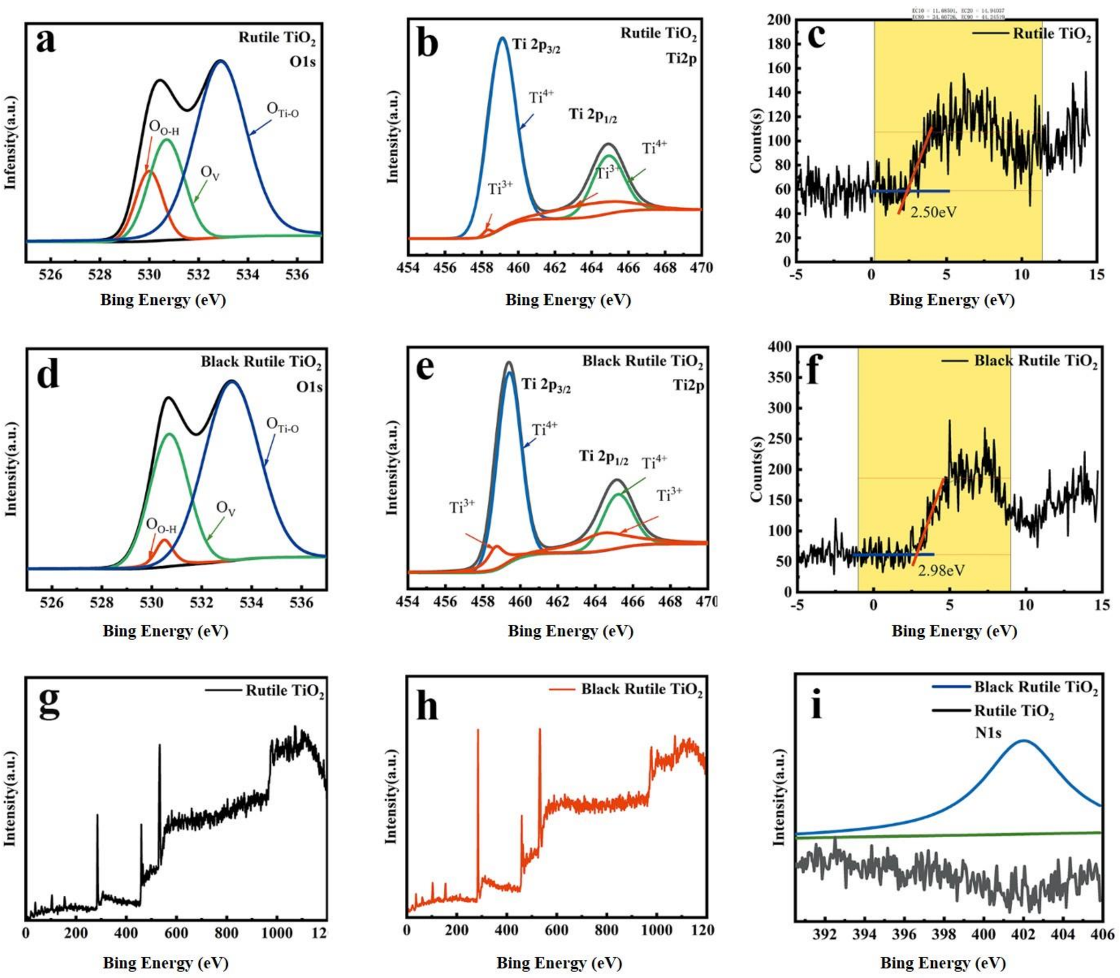

Figure S4. (**a**) O1s XPS spectrum of rutile TiO_2. (**b**) Ti2p XPS spectrum of rutile TiO_2. (**c**) UPS spectrum of rutile TiO_2. (**d**) O1s XPS spectrum of black rutile TiO_2. (**e**) Ti2p XPS spectrum of black rutile TiO_2. (**f**) UPS spectrum of black rutile TiO_2. (**g**) XPS survey of rutile TiO_2. (**h**) XPS survey of black rutile TiO_2. (**i**) N1s XPS spectrum of rutile TiO_2 and black rutile TiO_2. The green line is the baseline of the curve.

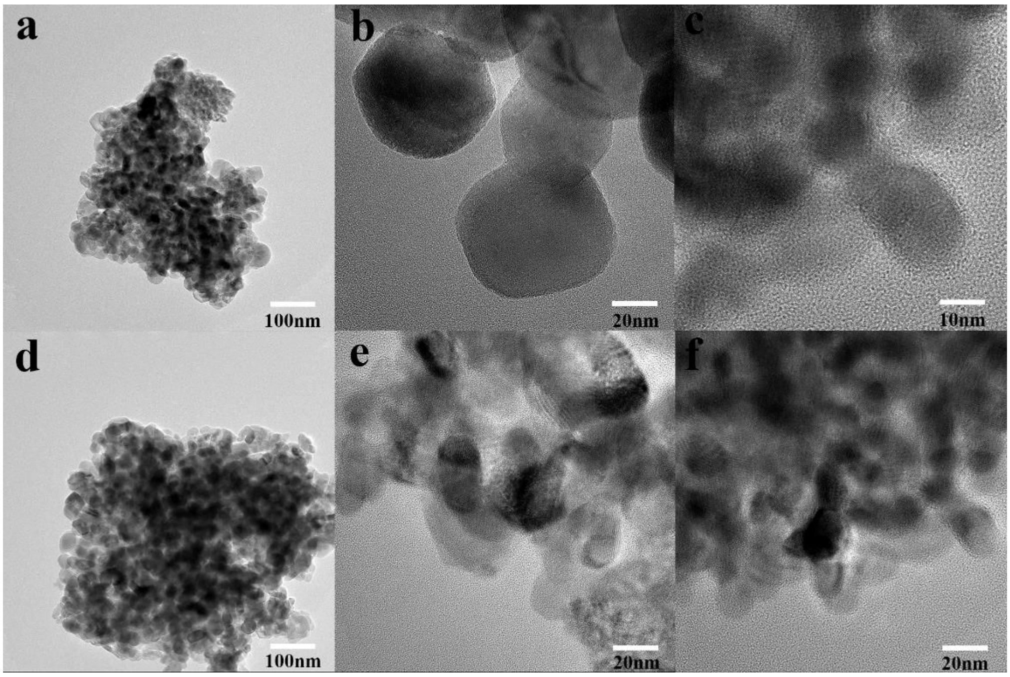

Figure S5. TEM and HRTEM images of anatase TiO_2 with different magnifications (**a**-**f**).

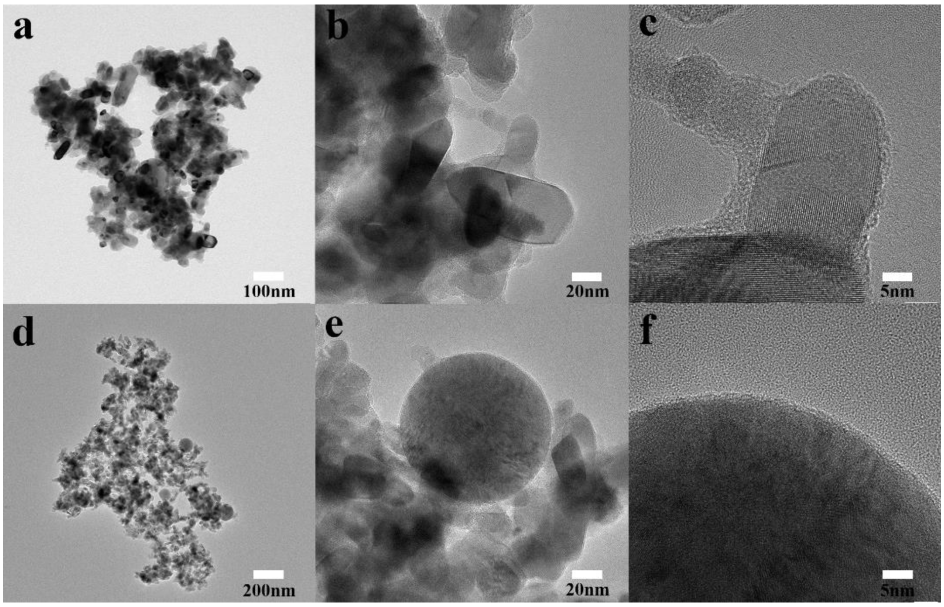

Figure S6. TEM and HRTEM images of black anatase TiO_2 with different magnifications (**a**-**f**).

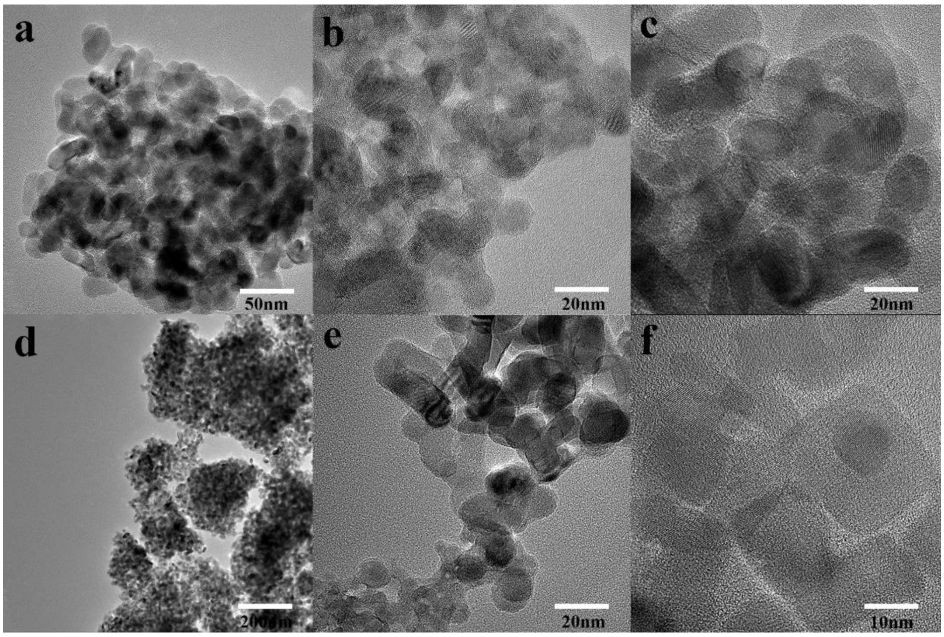

Figure S7. TEM and HRTEM images of rutile TiO_2 with different magnifications (**a**-**f**).

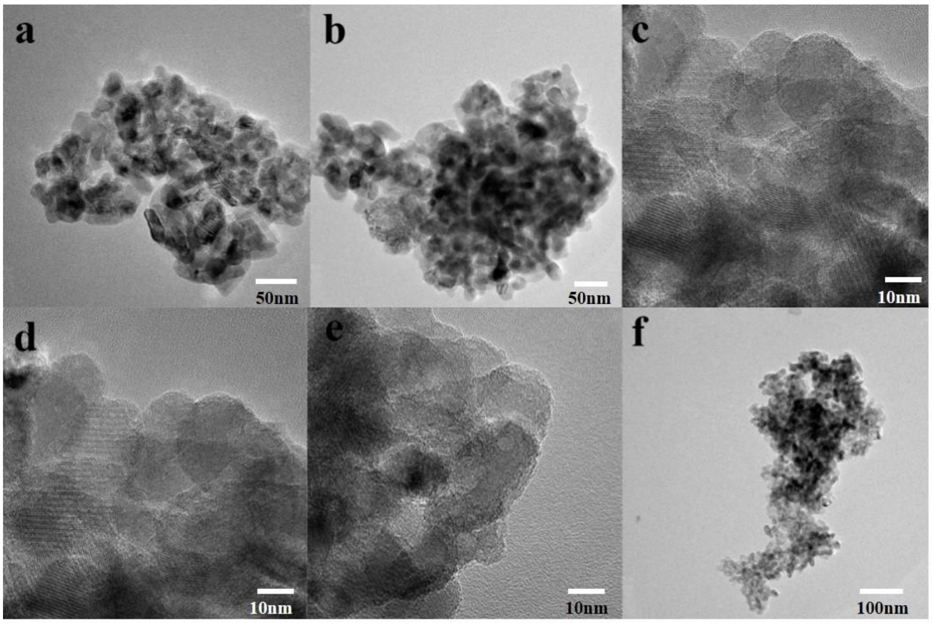

Figure S8. TEM and HRTEM images of black rutile TiO_2 with different magnifications (**a**-**f**).

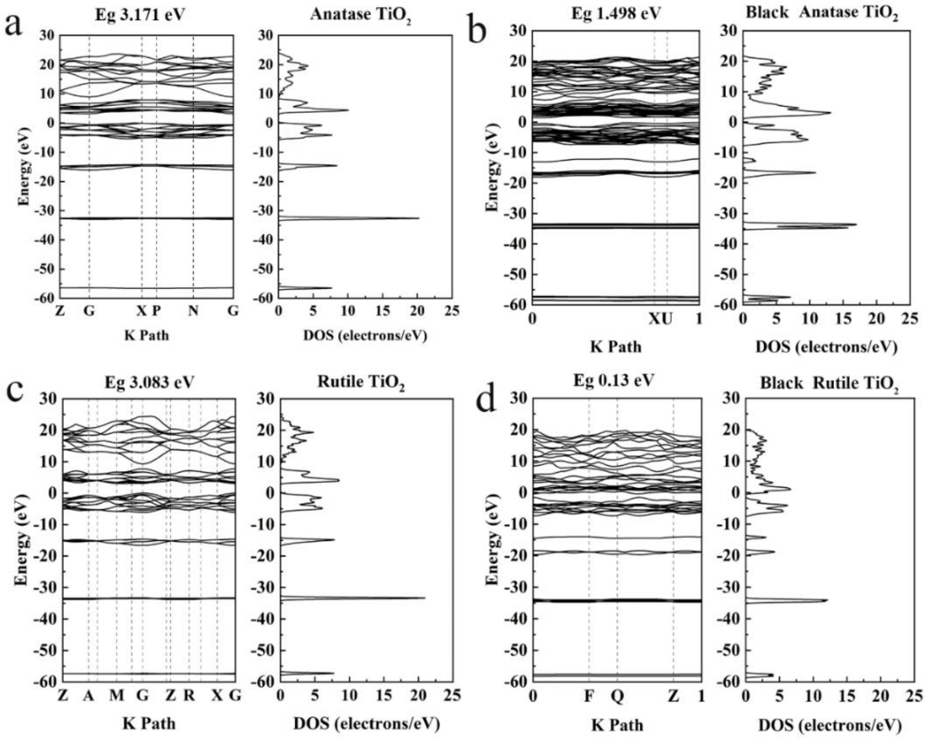

Figure S9. Energy band diagram and density of states spectrum obtained by first-principles calculations for (a) anatase TiO_2, (b) black anatase TiO_2, (c) rutile TiO_2, and (d) black rutile TiO_2.

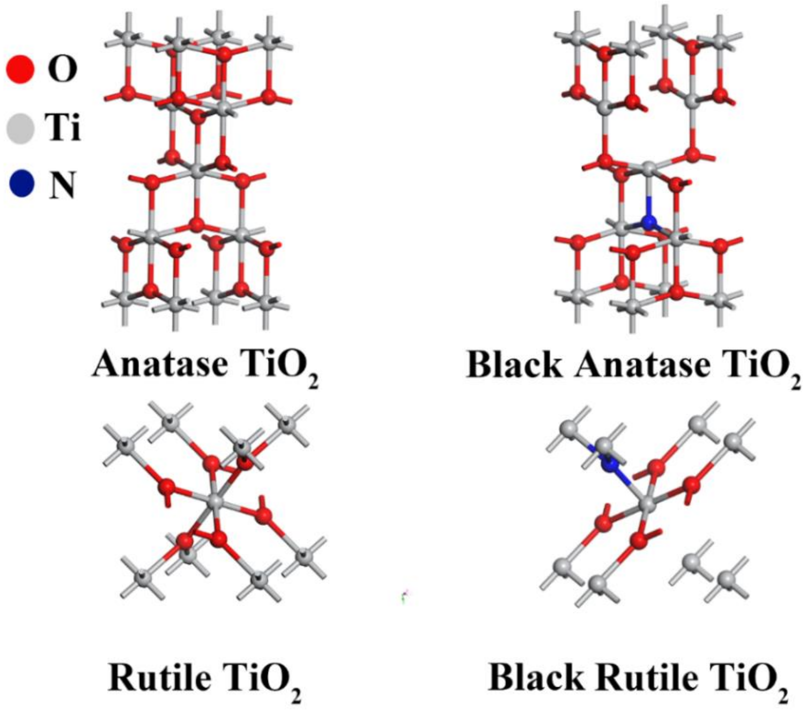

Figure S10. Unit cells of anatase TiO_2, black anatase TiO_2, rutile TiO_2, and black rutile TiO_2 for first-principles calculations.

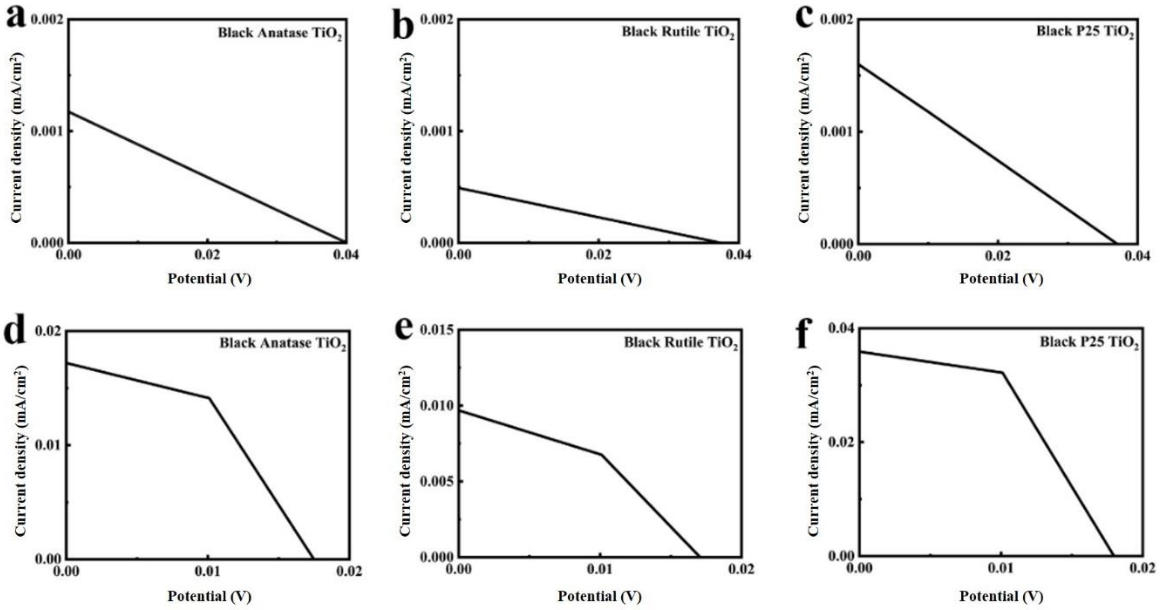

Figure S11. (**a**), (**b**) and (**c**) are J-V curves of black anatase TiO_2, rutile TiO_2, and P25 TiO_2 samples assembled with a S^{2-}/Sn^{2-} electrolyte and copper sulfide counter electrode without quantum dot sensitization. (**d**), (**e**) and (**f**) are J-V curves of black anatase TiO_2, rutile TiO_2, and P25 TiO_2 samples assembled with a platinum electrode using S^{2-}/Sn^{2-} electrolyte without quantum dot sensitization.

Table S1. Performance parameters of CdS/CdSe co-sensitized QDSSCs based on different reports.

Photoanode	QDs	J_{sc}(mA/cm^2)	V_{oc}(V)	FF	PCE(%)	Ref.
TiO_2 NPs	CdS/CdSe	11.91	0.59	0.51	3.56	S1
ZnO NDs/TiO_2 NPs	CdS/CdSe	15.34	0.66	0.53	5.36	S2
TiO_2 NWs–ZnO NSs	CdS/CdSe	16.11	0.51	0.55	4.57	S3
TiO_2 NWsTiO$_2$ NSs–ZnO NRs	CdS/CdSe	19.19	0.52	0.54	5.38	S4
TiO_2 MPsNWs	CdS/CdSe	19.32	0.53	0.59	6.01	S5
TiO_2 NWs	CdS/CdSe	17.98	0.47	0.50	4.20	S6
ZnO NDs	CdS/CdSe	16.0	0.62	0.49	4.86	S7
ZnO TP	CdS/CdSe	13.85	0.72	0.42	4.24	S8
ZnO NWs	CdS/CdSe	17.3	0.63	0.38	4.15	S9
TiO_2/ZnO NSs	CdS/CdSe	16.11	0.51	0.55	4.57	S10
black P25 TiO_2 NDs	CdS/CdSe	25.0	0.61	0.38	5.91	**This work**
D-G black P25 TiO_2 NDs	CdS/CdSe	50.**3**	0.61	0.39	11.67	**This work**

References

S1. Kim, S.K.; Raj, C.J.; Kim, H.J. CdS/CdSe quantum dot-sensitized solar cells based on ZnO nanoparticle/nanorod composite electrodes. *Electron. Mater. Lett.* **2014**, *10*, 1137–1142. https://doi.org/10.1007/s13391-014-4144-0.

S2. Jin, B.B.; Wang, Y.F.; Zeng, J.H. Performance enhancement in titania based quantum dot sensitized solar cells through incorporation of disc shaped ZnO nanoparticles into photoanode. *Chem. Phys. Lett.* **2016**, *660*, 76–80. https://doi.org/10.1016/j.cplett.2016.08.009.

S3. Feng, H.L.; Wu, W.Q.; Rao, H.S.; Wan, Q.; Li, L.B.; Kuang, D.B.; Su, C.Y. Three-dimensional TiO_2/ZnO hybrid array as a heterostructured anode for efficient quantum-dot-sensitized solar cells. *ACS Appl. Mater. Interfaces* **2015**, *7*, 5199–5205. https://doi.org/10.1021/am507983y.
S4. Feng, H.L.; Wu, W.Q.; Rao, H.S.; Li, L.B.; Kuang, D.B.; Su, C.Y. Three-dimensional hyperbranched TiO_2/ZnO heterostructured arrays for efficient quantum dot-sensitized solar cells. *J. Mater. Chem. A* **2015**, *3*, 14826–14832. https://doi.org/10.1039/C5TA02269J.
S5. Xu, Y.F.; Wu, W.Q.; Rao, H.S.; Chen, H.Y.; Kuang, D.B.; Su, C.Y. CdS/CdSe co-sensitized TiO_2 nanowire-coated hollow Spheres exceeding 6% photovoltaic performance.*Nano Energy* **2015**, *11*, 621–630. https://doi.org/10.1016/j.nanoen.2014.11.045.
S6. Kim, S.K.; Son, M.K.; Park, S.; Jeong, M.S.; Prabakar, K.; Kim, H.J. Surface modification on TiO_2 nanoparticles in CdS/CdSe Quantum Dot-sensitized Solar Cell. *Electrochim. Acta* **2014**, *118*, 118–123. https://doi.org/10.1016/j.electacta.2013.11.191.
S7. Raj, C.J.; Karthick, S.N.; Hemalatha, K.V.; Kim, H.J.; Prabakar, K. Highly efficient ZnO porous nanostructure for CdS/CdSe quantum dot sensitized solar cell. *Thin Solid Films* **2013**, *548*, 636–640. https://doi.org/10.1016/j.tsf.2013.10.009.
S8. Seol, M.; Kim, H.; Tak, Y.; Yong, K. Novel nanowire array based highly efficient quantum dot sensitized solar cell. *Chem Commun (Camb)* **2010**, *46*, 5521–5523. https://doi.org/10.1039/c0cc00542h.
S9. Kim, S.K.; Gopi C.V.V.M.; Rao, S.S.; Punnoose, D.; Kim, H.J. Highly efficient yttrium-doped ZnO nanorods for quantum dot-sensitized solar cells. *Appl. Surf. Sci.* **2016**, *365*, 136–142. https://doi.org/10.1016/j.apsusc.2016.01.043.
S10. Zhao, H.; Huang, F.; Hou, J.; Liu, Z.; Wu, Q.; Cao, H.; Jing, Q.; Peng, S.; Cao, G. Efficiency Enhancement of Quantum Dot Sensitized TiO_2/ZnO Nanorod Arrays Solar Cells by Plasmonic Ag Nanoparticles. *ACS Appl. Mater. Interfaces* **2016**, *8*, 26675–26682. https://doi.org/10.1021/acsami.6b06386.
